# High and Low Media Multitaskers Differ on Cued But Not Voluntary Task Switching

**DOI:** 10.1027/1618-3169/a000639

**Published:** 2025-04-23

**Authors:** Jackson S. Colvett, L. Casey Bales, Janine M. Jennings

**Affiliations:** ^1^ Department of Psychological Science, Berry College, Mt Berry, GA, USA; ^2^ Department of Psychology, Wake Forest University, Winston-Salem, NC, USA

**Keywords:** media multitasking, task switching, voluntary task switching

## Abstract

**Abstract:** Media multitasking (i.e., the use of multiple forms of media at the same time) is an increasingly common behavior. As media multitasking requires switching between different forms of media, there has been great interest in its relationship with the ability to switch between tasks. Clear patterns have not emerged in cued task switching, as studies have found that high media multitaskers switch more effectively, switch less effectively, or that there are no differences between high and low media multitaskers. The voluntary task switching paradigm provides an alternate and yet unexplored perspective that could reveal differences between high and low media multitaskers in terms of how effectively and how often they switch. In Experiment 1, high media multitaskers had a smaller cued task switching switch cost, but no difference in voluntary switch cost or switch rate. Experiment 2 explored whether voluntary task switching differences emerged at longer response stimulus intervals (RSIs). Again, no group difference was observed in voluntary switch cost or switch rate. We discuss the differences between what is assessed in cued and voluntary task switching paradigms and subsequent implications for media multitasking.


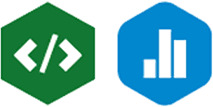


Sitting down to read a research article, would you answer an email, check your cellphone, or play music in the background? These behaviors describe *media multitasking*, the use of multiple types of media at the same time. As devices such as computers and smartphones have become more mobile and accessible, media consumption has increased (Aagaard, 2015; Baumgartner et al., 2014), and media multitasking has emerged as an increasingly common set of behaviors. In 2024, adults in the United States reported more media use than any previous year, and the nature of that media use has become increasingly digital and phone-based (Cramer-Flood et al., 2024). As media multitasking behavior has increased, so has research interest in the cognitive profile of frequent (high media multitaskers, HMMs) and infrequent (low media multitaskers, LMMs) media multitaskers. Importantly, some media multitasking is cued by an external signal from the environment, like a phone notification drawing you away from reading. Other media multitasking is driven by an internal desire to switch, like looking away from a movie and toward your phone during a scene you find particularly boring. In the current study, we aimed to better understand the cognitive abilities of high and low media multitaskers by considering both externally and internally cued task switching.

The dominant approach to understanding the relationship between media multitasking and cognition has been to assess media multitasking frequency via questionnaire, typically using the Media Multitasking Index (MMI, Ophir et al., 2009), then examine whether self-reported HMMs and LMMs differ on a given cognitive task. Note that this approach provides no directionally causal claim; a person high (or low) in a cognitive ability score may be more likely to media multitask, experience with media multitasking may improve (or harm) cognitive ability, or both could be true. In addition to the MMI, participants in the initial study (Ophir et al., 2009) completed a battery of cognitive tests. HMMs performed worse than LMMs specifically on tasks that stress the ability to filter interfering information (i.e., an irrelevant shape stimulus filtering task, AX-CPT with distractors, n-back task, and cued task switching). Since the initial study, a literature has emerged comparing HMMs and LMMs across a wide array of cognitive tasks (see, Uncapher & Wagner, 2018; Parry & le Roux, 2021; Wiradhany & Nieuwenstein, 2017, for reviews).^1^ As real-world media multitasking requires people to switch between multiple forms of media, research interest has been particularly drawn toward whether HMMs and LMMs differ in their ability to switch between tasks.

## Media Multitasking and Task Switching

Commonly, task switching ability is measured by *switch costs* or the difference in performance in reaction time or error rate between trials where the task is the same as the previous trial (i.e., repeat trial) and trials where the task differs from the previous trial (i.e., switch trial). A larger switch cost suggests a difficulty in inhibiting the previous task set and switching to the current one. Ophir et al. (2009) compared HMMs and LMMs in a cued task switching paradigm where participants determined if a stimulus was a consonant or vowel when cued with *LETTER* or if the stimulus was even or odd when cued with *NUMBER*. After the cue, participants were presented a bivalent stimulus consisting of a number and a letter (e.g., “2b”), and asked to attend and respond to the stimulus corresponding with the cue. HMMs showed larger switch costs than LMMs (Ophir et al., 2009), which the authors attributed to HMMs having less effective top-down attentional processing and thus greater difficulty filtering irrelevant information from memory (e.g., the irrelevant task set). A subsequent replication of the Ophir et al. (2009) study found that the larger switch costs for HMMs were driven by greater interference on switch trials for the HMM group rather than differences between groups on repeat trials (Wiradhany & Nieuwenstein, 2017). Relatedly, HMMs have been found to be particularly susceptible to interference from irrelevant information in the form of distractors in an AX-CPT task (Cardoso-Leite et al., 2016; Ophir et al., 2009).

However, it is not universally found that HMMs have larger switch costs than LMMs. Other studies have found no difference in switch costs between media multitasking groups (Cardoso-Leite et al., 2016; Gorman & Green, 2016; Minear et al., 2013; Schneider & Chun, 2021) or have even found that HMMs switch more effectively than LMMs (Alzahabi & Becker, 2013; Alzahabi et al., 2017; Elbe et al., 2019). Alzahabi and Becker (2013) replicated the methods of Ophir et al. (2009) but found that HMMs had *smaller* switch costs in reaction time than LMMs. They argued that HMMs reconfigure tasks more efficiently on a switch trial because media multitasking serves as a form of cognitive training that generalizes to rapid reconfiguration of task sets during lab-based task switching. Alzahabi and Becker (2013) also note that the ubiquity and nature of media multitasking has changed since Ophir et al. (2009) ran their initial experiment. That trend in media multitasking has likely continued, as overall media use continues to increase (e.g., Cramer-Flood et al., 2024; V. Rideout, 2016; V. Rideout & Robb, 2019).

To better understand these disparate results, recent work has attempted to understand which elements of task switching relate to media multitasking. Alzahabi et al. (2017) replicated the result that HMMs switched more effectively than LMMs and found that media multitasking frequency correlated most strongly with reconfiguring and preparing a new task set. Relatedly, when participants switched between tasks using different tabs on a computer, media multitasking differences depended on whether participants could freely choose which tab they were using (Szumowska et al., 2018). HMMs switched more often, albeit less effectively, than LMMs when participants freely chose to switch between tabs (i.e., internally driven switching) while no differences emerged when switches were cued sequentially by the experiment (i.e., externally driven switching). Additionally, in a paradigm where participants were allowed to select the order of trials from two tasks on an upcoming block, HMMs were more likely to select switches between tasks and LMMs were more likely to select blocked sequences with fewer switches between tasks (Gaujoux et al., 2022). Relatedly, HMMs were more likely to select blocks with a high proportion of switch trials when given the opportunity to select blocks with 100%, 75%, 25%, or 0% switches (Gaujoux et al., 2024). If HMMs perform differently than LMMs in situations that prioritize internal preparation of a task set, important relationships to media multitasking may be found using an index of self-directed task set preparation: the Voluntary Task Switching (VTS) paradigm.

## Voluntary Task Switching

The VTS paradigm (Arrington & Logan, 2004; see Arrington et al., 2014 for a review) was developed in response to debates within the cued task switching (CTS) literature as to whether switch costs reflected active, top-down reconfiguration of task sets or were instead driven by bottom-up information from the stimuli or task context (Arrington & Logan, 2005). During VTS, a multivalent stimulus (e.g., participants could either judge that the number 4 is even or that it is greater than 3) is presented on screen. There is no external cue telling participants which task to perform, so participants decide whether to repeat the task from the previous trial or switch to another task. Each task has a separate set of responses (i.e., press “d” and “f” keys for even and odd; press “j” and “k” keys for higher or lower) that participants use to respond with for the task they choose on that trial. We focus on VTS paradigms with this response type as it is used in the current study, though it should be noted that other VTS paradigms have participants register a task choice before stimulus onset then use shared responses for each task (e.g., Kessler et al., 2009; Mittelstädt et al., 2018). In VTS, participants are typically instructed to choose a task as if the decision was made randomly on each trial, and to try to perform each task on 50% of trials.

As previous work on media multitasking has only used CTS, VTS offers a different perspective for examining the relationship between task switching and media multitasking. In everyday life, media multitasking is driven by both external and internal cues. Though some media switches are undoubtably cued externally (e.g., email notification, phone vibration), other switches are driven by internal motivations to use a different type of media. Moreover, media multitasking may relate to elements of task switching that are not present in typical CTS paradigms, such as self-directing the preparation of new task sets and inhibition of old task sets in the absence of an external cue. Using the VTS in addition to the CTS paradigm may capture differences in internally driven media multitasking behavior between HMMs and LMMs in a way that CTS alone does not.

## Measuring Media Multitasking

To assess the relationship between media multitasking and task switching, some measure of media multitasking must be used to differentiate high from low media multitaskers. The majority of studies have used the media multitasking index (MMI; Ophir et al., 2009) to assess *the proportion of media time* spent multitasking. However, the MMI as a measure and the literature’s ability to differentiate high from low media multitasking is not without criticism (see Parry & Fisher, 2024). In short, the MMI is exceedingly lengthy (i.e., 144 questions for the common version assessing 12 media types), fails to capture many newer media types, and focuses on proportion of media time media multitasking rather than the potentially also important total time spent media multitasking. In addition to collecting the MMI for each participant, we devised the Media Multitasking Engagement Survey (MMES) as a complementary and alternative measure of media multitasking to address some of these potential issues with the MMI. The MMES^2^ was adapted in part from the media multitasking measure used by Xu et al. (2016) while also adding more recent forms of media such as social media and video chatting. The survey was designed to determine the approximate amount of time spent with 15 types of media on a typical day and estimate the *amount of time spent media multitasking in hours* with each type of media. Importantly, we still collected MMI scores to provide appropriate comparison to other media multitasking studies and ensure our HMM and LMM groups also differed in terms of the MMI.

## Current Study

The current work was designed to examine differences in VTS performance among high and low media multitaskers. As switch costs in VTS capture different elements of task switching than CTS, and VTS allows an assessment of switch rate, we wondered if differences between HMMs and LMMs might emerge that were not captured by previous studies. We conducted two experiments to investigate the relationship between media multitasking and VTS. Experiment 1 replicated and extended previous work assessing the relationship between media multitasking and task switching by including both the CTS and VTS paradigms. We found that HMMs switched more effectively than LMMs in CTS, but no difference emerged in VTS. An important factor to consider in Experiment 1 was our chosen response stimulus interval (RSI), or the time between previous response and subsequent stimulus. As VTS performance has different profiles at short and long RSIs (e.g., Arrington, 2008; Arrington & Logan, 2004, 2005; Liefooghe et al., 2010), we wanted to assess whether the null difference in Experiment 1 was driven by the design choice of a 100 ms RSI. Experiment 2 only assessed VTS but included conditions with a short (100 ms) or long (1,000 ms) delay between trials. Again, no difference was found between media multitasking groups in VTS. The pattern that emerged indicated a relationship between media multitasking and task switching, but specifically for elements of task switching that relate to goal-relevant external cue use. We discuss implications of both studies, and how they inform our understanding of media multitasking and different task switching paradigms.

Finally, previous work has demonstrated that voluntary switching is not exclusively endogenous; participants are more likely to choose to repeat a task when the stimulus is the same as the previous trial (i.e., stimulus repetition) than if the stimulus differs from the previous trial (i.e., stimulus change; e.g., Mayr & Bell, 2006). While bottom-up signals from cue repetition have been shown not to differentially affect HMMs and LMMs (Schneider & Chun, 2021 note that this study also found no difference between HMMs and LMMs on trials were the cue did not repeat), we still assessed the effect of a stimulus repetition on performance. More specifically, we assessed whether stimulus repetitions and stimulus changes affected switch rate and switch costs differentially for HMMs and LMMs. A full report of our stimulus repetition analyses is available in the Electronic Supplementary Materials, ESM 1. In short, we replicated typical findings (participants were more likely to switch tasks and switch costs were lower on stimulus switches than stimulus repetitions), but we found no difference between HMMs and LMMs in terms of sensitivity to stimulus-based switching for either experiment.

## Experiment 1

The first key question of Experiment 1 was whether there would be a relationship between media multitasking and CTS performance. In keeping with previous results demonstrating smaller cued switch costs for HMMs (e.g., Alzahabi & Becker, 2013; Alzahabi et al., 2017; Elbe et al., 2019), we expected HMMs to have smaller switch costs in both RT and error rate. The second key question of the current study entailed whether HMMs differ from LMMs in terms of VTS performance. We expected that HMMs would switch more effectively (i.e., a smaller switch cost) and more often (i.e., a higher switch rate) than LMMs based on previous work demonstrating that HMMs had smaller switch costs than LMMs in CTS paradigms that emphasized preparation of the task set (Alzahabi et al., 2017). In terms of switch rate, HMMs have been shown to switch between sources of information more when they had freedom to choose (Szumowska et al., 2018). We predicted that HMMs would have a higher switch rate than LMMs, but both groups will switch less often than 50% of trials as is expected in voluntary task switching (Arrington & Logan, 2004, 2005; Mittelstädt et al., 2018; Yeung, 2010).

### Methods

#### Participants

Forty-four undergraduates (19 females, 25 males; age *M* = 19.18 years, *SD* = 1.11) participated and received course credit in exchange for their participation. This sample size was selected because it was comparable to previous studies comparing media multitasking and CTS (Ophir et al., 2009; *N* = 30; *t* = 2.62 comparing switch costs between HMM and LMM) as well as previous VTS studies (Arrington & Logan, 2004; *N* = 17; η_*p*_^2^ = .85 comparing voluntary repetitions to voluntary switches; Yeung, 2010; *N* = 16; *t* = 12.9 comparing switch rate to .50). Two hundred and fifty-four undergraduate students completed the MMES and MMI among a set of surveys to assess eligibility to participate in various studies for an undergraduate research pool. Participants scoring in the middle quartiles of the MMES or who were diagnosed with ADD or ADHD were excluded from participation. Individuals scoring in the top or bottom quartiles of the MMES were invited to participate.

Twenty-two participants scoring in the top quartile of the MMES (12 females, 10 males; age *M* = 19.00 years, *SD* = .87; MMES score *M* = 16.86, *SD* = 6.63) comprised the HMM group. Twenty-two participants scoring in the bottom quartile of the MMES (7 females, 15 males; age *M* = 19.36 years, *SD* = 1.29; MMES score *M* = 3.17, *SD* = 1.03) comprised the LMM group. All participants were within 3 *SD* of their group’s mean. As expected, HMMs (*M* = 4.65, *SE* = 0.31) scored significantly higher than LMMs (*M* = 2.74, *SE* = 0.93) on the MMI, *t*(42) = 5.16, *p* < .001, *d =* 1.56.

#### Materials and Procedure

The behavioral task switching paradigms were completed on laptops running E-Prime Pro 2.0 (Schneider et al., 2002), and a series of self-report measures were administered using Qualtrics (2012). Task switching procedures were adapted from Mayr and Bell (2006). Participants were tested one at a time with an experimenter present throughout the study. Participants performed two number judgment tasks using a laptop computer. A number between 1 and 5, excluding 3, was presented centrally in black on a white background. Participants judged whether the number was higher or lower than three or if the number was odd or even. As in Mayr and Bell (2006), tasks were mapped to separate hands. Participants used their right middle and index finger to press “j” and “k” and their left middle and index finger to press “d” and “f” to give answers for each task. Task to hand response mapping was counterbalanced across participants. A new stimulus was presented 100 ms following a participant’s response.

Participants completed 12 48-trial blocks. For each change in block type, instructions were presented on the screen and participants performed a 16-trial practice block which they had the option of repeating if they believed they needed additional practice. The first four blocks consisted of single task performance (lower/higher task for Blocks 1 and 2; odd/even task for Blocks 3 and 4). Blocks 5–8 were VTS. We presented VTS blocks before CTS blocks for all participants because we did not want the 50% switch rate in CTS to impact participants’ task choice during a subsequent VTS block (Fröber & Dreisbach, 2017; c.f. Fröber et al., 2022). Participants were told they were to mix together the two previous tasks, and they should perform each task on about half of the trials. They were then told to perform the tasks in a random order “as if their decision was determined by a coin toss” (see Arrington & Logan, 2005), rather than a nonrandom order such as alternating between tasks. Participants were also asked not to keep track of how many times they had performed each task. The experimenter watched and reminded the participants of the instructions if a participant was responding with a noticeable pattern or did not switch between tasks. Blocks 9–12 were CTS. In addition to the number stimuli, a cue was presented above the stimuli (e.g., either *Lower/Higher* or *Even/Odd*) telling which participant to perform on that trial. This cue appeared concurrently with the number stimulus and was presented above the number. The cue and stimulus remained on screen until response. The tasks were presented in an AABB pattern, such that a participant would perform the magnitude judgment task for two sequential trials (a repeat trial) followed by a switch trial where they performed the parity judgment task and then repeated it. Participants were not explicitly informed about the AABB task order. The experiment took around 30 min to administer.

### Results

To account for outliers in task switching, median RT for participant performance in each condition was used. Only correct trials were retained in the analysis of RTs. See Tables 1 and 2 for condition means.

**Table 1:** Experiment 1: Single task reaction times and error rates

Media multitasking group	Trial type	Reaction time (ms)	Error rate (%)
HMM	High/low	454 (15)	2.0 (0.46)
	Even/odd	474 (13)	5.1 (0.68)
LMM	High/Low	435 (11)	2.3 (0.47)
	Even/odd	450 (11)	5.3 (0.74)
*Note*. HMM = high media multitaskers; LMM = low media multitaskers. The reaction time column represents the condition mean of all participants’ median reaction times.			

**Table 2:** Experiment 1: Cued and voluntary task switching reaction times and error rates

Task	Media multitasking group	Trial type	Reaction time (ms)	Error rate (%)
Cued task switching	HMM	Repeat	727 (23)	6.5 (1.14)
		Switch	1,148 (46)	9.1 (1.57)
	LMM	Repeat	709 (20)	5.6 (0.97)
		Switch	1,253 (45)	9.7 (1.82)
Voluntary task switching	HMM	Repeat	577 (37)	4.5 (0.64)
		Switch	934 (36)	8.0 (1.46)
	LMM	Repeat	523 (32)	3.7 (0.81)
		Switch	947 (54)	5.7 (1.31)
*Note*. HMM = high media multitaskers; LMM = low media multitaskers. The reaction time column represents the condition mean of all participants’ median reaction times.				

We additionally report Bayesian evidence for the null hypothesis compared to evidence for the alternative hypothesis (BF_01_), such that larger numbers indicate relatively stronger evidence for the null. A value between 1 and 3 indicates anecdotal evidence for the null hypothesis and a value between 3 and 10 indicates substantial evidence for the null hypothesis (Wagenmakers et al., 2011). Analyses were run using the baseline settings^3^ of JASP Version 0.17.1 (JASP Team, 2024; Van Doorn et al., 2020).

For performance in the single task conditions, we performed an independent samples *t*-test, demonstrating nonsignificant group differences in RTs for the higher/lower task [*t*(42) = 0.99, *p =* .330 , *d* = .30, BF_01_ = 2.28] or the odd/even task [*t*(42) = 1.41, *p =* .167, *d* = .42, BF_01_ = 1.52]. We also found no difference in error rate for the higher/lower task [*t*(42) = .56, *p =* .582, *d* = .17, BF_01_ = 2.97] or the odd/even task [*t*(42) = 0.18, *p =* .857 , *d* = .05, BF_01_ = 3.32].

#### Cued Task Switching

**Figure 1:**
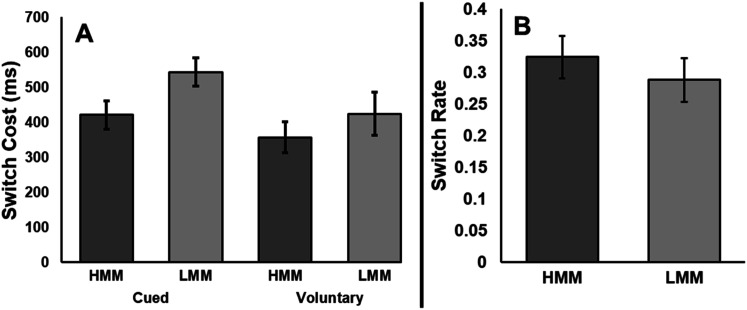
Experiment 1 results for reaction time switch costs for CTS and VTS (Panel A) and VTS switch rate (Panel B). Error bars depict standard errors. Switch costs were significantly smaller for HMMs than LMMs in CTS. However, no difference in VTS switch cost or switch rate emerged between media multitasking groups.

We conducted 2 x 2 mixed effects ANOVAs for both RT and error rate in CTS. Media multitasking group (HMM vs. LMM) was between-subjects and trial type (repeat vs. switch) was within-subjects. For RT, responses on repeat trials (*M* = 718, *SE* = 15) were significantly faster than responses on switch trials (*M* = 1,200, *SE* = 32), *F*(1, 42) = 303.50, *p* < .001, η_*p*_^2^ = .88, BF_01_ < .001, regardless of group. While the media multitasking groups did not significantly differ [*F*(1, 42) = 1.06, *p* = .309, η_*p*_^2^ = .03, BF_01_ = 2.25], trial type and media multitasking group significantly interacted, *F*(1, 42) = 4.98, *p* = .031, η_*p*_^2^ = .11, BF_01_ = 0.43, such that switch costs were smaller for HMMs (*M* = 421, *SE* = 40) than LMMs (*M* = 544, *SE* = 41; see Figure 1).

Participants made significantly fewer errors on repeat trials (*M* = 6.00%, *SE* = 0.75%) than switch trials (*M* = 9.41%, *SE* = 1.20%), *F*(1, 42) = 22.04, *p* < .001, η_*p*_^2^ = .34, BF_01_ < .001. There was not a significant difference between media multitasking groups in overall error rate [*F*(1, 42) = 0.01, *p* = .942, η_*p*_^2^ < .01, BF_01_ = 2.16] nor did group and trial type significantly interact [*F*(1, 42) = 1.13, *p* = .293, η_*p*_^2^ = .03, BF_01_ = 2.15].

#### Voluntary Task Switching

For both RT and error rate in VTS blocks, 2 x 2 mixed effects ANOVAs were conducted. Media multitasking group (HMM and LMM) was between-subjects, and trial type (repeat and switch) was within-subjects. Repeat trials (*M* = 550, *SE* = 25) were significantly faster than switch trials (*M* = 941, *SE* = 32), *F*(1, 42) = 110.32, *p* < .001, η_*p*_^2^ = .72, BF_01_ < .001. Additionally, there was no effect of media multitasking group, *F*(1, 42) = 0.22, *p* = .640, η_*p*_^2^ = .01, BF_01_ = 3.47. Unlike CTS, trial type and media multitasking group did not interact [*F*(1, 42) = 0.81, *p* = .374, η_*p*_^2^ = .02, BF_01_ = 2.39] indicating no significant difference between the switch costs of HMMs and LMMs (see Figure 1).

For error rate, responses to repeat trials (*M* = 4.07%, *SE* = 0.52%) were more accurate than switch trials (*M* = 6.82%, *SE* = 0.99%), *F*(1, 42) = 11.69, *p* = .001, η_*p*_^2^ = .22, BF_01_ = .04. Media multitasking groups did not significantly differ, *F*(1, 42) = 1.28, *p* = .26, η_*p*_^2^ = .03, BF_01_ = 1.86, nor was there a significant interaction between trial type and media multitasking group, *F*(1, 42) = 0.87, *p* = .360, η_*p*_^2^ = .02, BF_01_ = 2.41.

We also assessed whether the switch rate differed between HMMs and LMMs. Because participants choose which task to perform on each trial, switch rate indicates a participant’s flexibility and willingness to switch tasks. Note that a participant who perfectly followed the instructions would have a switch rate of 50%, though participants typically switch less often (e.g., Arrington & Logan, 2004, 2005; Mittelstädt et al., 2018; Yeung, 2010). Using a one-sample *t*-test, we found that the participants’ switch rate (*M* = 30.58%, *SE* = 2.44%) was significantly less than 50% (*t*(43) = 8.16, *p* < .001, *d* = 1.23, BF_01_ < .001). An independent samples *t*-test revealed that switch rate did not significantly differ between HMMs and LMMs, *t*(42) = 0.75, *p =* .456, *d* = .23, BF_01_ = 2.68 (see Figure 1).

### Discussion

In Experiment 1, distinct patterns emerged between HMMs and LMMs in CTS and VTS. HMMs had smaller switch costs than LMMs when cued, replicating the effect observed in some studies (Alzahabi & Becker, 2013; Alzahabi et al., 2017) but not others (Ophir et al., 2009; Wiradhany & Nieuwenstein, 2017). We found strong evidence that HMMs switched more effectively than LMMs, such that the effect size (η_*p*_^2^ = .11) indicated a medium to large interaction between media multitasking group and switch cost and the Bayes Factor indicated substantial evidence in favor of the interaction over the null (BF_01_ = 0.43). Previous studies have postulated about negative effects of media multitasking. While the design of the current study does not allow us to endorse a causal or directional claim about media multitasking improving cued task switching ability, our finding that HMMs switched more effectively than LMMs in CTS would be inconsistent with an account that proposed a deleterious effect of media multitasking. We believe it is plausible that people who are more susceptible to cued switch costs avoid media multitasking in real life (i.e., LMMs). Indeed, metacognitive awareness about switching and distraction has been shown to affect participants choice to media multitask in a work context (Whittaker et al., 2016).

In terms of the processes underlying task switching, this result indicates that HMMs may reconfigure task sets more effectively when cued to switch. One should note the AABB pattern used by Mayr and Bell (2006) and our method makes the CTS paradigm predictable. Other CTS paradigms provide cues in a random or pseudorandom pattern, thus making the task on the next trial unpredictable. Compared to an unpredictable cue, a predictable cue plausibly allows for some internal preparation of a task set once a participant learns the pattern. As HMMs outperformed LMMs in a design that encouraged task set preparation (Alzahabi et al., 2017), it is possible that this predictable design choice may have played a role in us observing a difference between HMMs and LMMs in CTS.

One should also note that both HMMs and LMMs were slower in CTS blocks than in VTS blocks. There are several possible reasons for this difference. First, we presented the task cue concurrently with the stimulus. While participants could have learned the AABB pattern and not attended to the cue, we did not explicitly instruct participants to do so, nor did we explicitly make them aware of the AABB pattern. Additionally, nonoverlapping response sets were used for the two tasks (i.e., the magnitude and parity judgments were completed using different hands), which is a diversion from many CTS studies that use overlapping response sets. Finally, the cued task switching was always done after VTS, so it is possible that participants were more fatigued at that point of the experiment. As the magnitude of switch costs is larger when responses are slower overall, it is possible that the slower response time in CTS may have led to greater power to observe a significant difference between media multitasking groups in CTS than in VTS.

Although switch costs were nominally smaller for HMMs than for LMMs when switching voluntarily, the difference was not significant. Based on the results of Experiment 1, we cannot conclude that the endogenous nature of VTS more accurately captured the type of switching one can associate with real-world media multitasking. However, it is possible that the relatively short RSI used in Experiment 1 contributed to the null difference between HMMs and LMMs. We selected the 100 ms RSI because it was used in the previous VTS study (Mayr & Bell, 2006) upon which we modelled our procedure. When participants have more time to reconfigure the task set and prepare for the next trial, switch costs are reduced in CTS (e.g., Rogers & Monsell, 1995). Similarly, participants used a preparatory interval to reconfigure task sets in VTS. After a longer RSI, switch costs decreased (Arrington, 2008; Arrington & Logan, 2004, 2005; Liefooghe et al., 2010) and switch rate increased (Arrington & Logan, 2004, 2005; Demanet & Liefooghe, 2014). It is possible that HMMs do differ from LMMs in voluntary task switching, but only when they have enough time to reconfigure the task set between trials.

## Experiment 2

We manipulated RSI so participants performed in both short (100 ms) and long (1,000 ms) RSI conditions. As the focus of this experiment was to assess this difference in VTS, we did not include CTS blocks. If HMMs switch more often and more effectively with sufficiently long RSIs, we predict a higher switch rate and smaller switch costs for HMMs after a long RSI but no difference between groups after a short RSI.

### Method

#### Participants

Sixty-eight undergraduates (34 females; age *M* = 18.94 years, *SD* = 0.93) participated in exchange for course credit. Three hundred and sixty-five undergraduates initially completed the MMES and MMI as part of a set of surveys to assess eligibility to participate in various studies. Participants scoring in the middle quartiles of the MMES or who were diagnosed with ADD or ADHD were excluded from participation. Individuals scoring in the top or bottom quartiles on the MMES were invited to participate.

Thirty-five participants scoring in the top quartile of the MMES (20 females; age *M* = 18.94 years, *SD* = 1.14; MMES score *M* = 14.62, *SD* = 5.70) comprised the HMM group. Thirty-three participants scoring in the bottom quartile of the MMES (14 females; age *M* = 18.94 years, *SD* = 0.66; MMES score *M* = 2.16, *SD* = 1.17) comprised the LMM group. All participants were within 3 *SD* of their group’s mean. As in Experiment 1, HMMs (*M* = 4.36, *SE* = 1.31) also had significantly higher MMI scores than LMMs (*M* = 3.51, *SE* = 1.41, *t*(66) = 2.85, *p* = .006, *d =* 0.69.

#### Materials and Procedure

The voluntary task switching procedure for this study was identical to Experiment 1 except for a few key differences. First, participants were administered 10 48-trial blocks. In Blocks 1–2, participants completed the lower/higher task with an RSI of either 100 or 1,000 ms. Whichever RSI was not used in Block 1 was used in Block 2. In Blocks 3–4, participants completed the odd/even task with an RSI of either 100 or 1,000 ms. Whichever RSI was not used in Block 3 was used in Block 4. The order of the two single tasks was counterbalanced across participants. Blocks 5–10 assessed VTS. The RSIs used in each block followed an ABABAB pattern, such that a participant would perform the voluntary switching block with an RSI of 100 ms, followed by a block with an RSI of 1,000 ms. RSI order was counterbalanced across all participants. Participants were then administered the MMI (Ophir et al., 2009) and a demographics form asking their age and gender. This procedural step differed from Experiment 1, where the MMI was administered as part of the initial participant screening questionnaires. The entire experiment took around 30 min to administer.

### Results

Median RT for participant scores in each condition was used to correct for outliers and analyses of error rate only used correct responses. For each single task, we performed a mixed-factors ANOVA with the between-subjects factor of media multitasking group (HMM and LMM) and the within-subjects factor of RSI (100 ms and 1,000 ms). See Tables 3 and 4 for condition means.

**Table 3:** Experiment 2: Single task reaction times and error rates

Media multitasking group	RSI (ms)	Task	Reaction time (ms)	Error rate (%)
HMM	100	High/low	455 (9)	1.2 (0.25)
		Even/odd	472 (8)	2.9 (0.37)
	1,000	High/low	452 (9)	1.7 (0.38)
		Even/odd	489 (10)	3.3 (0.37)
LMM	100	High/low	467 (12)	2.0 (0.31)
		Even/odd	481 (13)	3.5 (0.42)
	1,000	High/low	450 (11)	2.0 (0.35)
		Even/odd	482 (12)	3.4 (0.48)
*Note*. HMM = high media multitaskers; LMM = low media multitaskers. RSI = response stimulus interval. The reaction time column represents the condition mean of all participants’ median reaction times.				

**Table 4:** Experiment 2: Voluntary task switching reaction times and error rates

Media multitasking group	RSI (ms)	Trial type	Reaction time (ms)	Error rate (%)
HMM	100	Repeat	568 (35)	2.4 (.35)
		Switch	935 (35)	4.8 (.73)
	1,000	Repeat	574 (16)	3.3 (.48)
		Switch	750 (36)	4.1 (.47)
LMM	100	Repeat	534 (33)	3.1 (.57)
		Switch	889 (32)	5.2 (.98)
	1,000	Repeat	549 (21)	3.4 (.63)
		Switch	687 (30)	3.6 (.68)
*Note*. HMM = high media multitaskers; LMM = high media multitaskers. RSI = response stimulus interval. The reaction time column represents the condition mean of all participants’ median reaction times.				

In the lower/higher task, reaction time did not significantly differ by RSI (*F*(1, 68) = 1.89, *p* = .174, η_*p*_^2^ = .03, BF_01_ = 2.55), or media multitasking group [*F*(1, 68) = 0.19, *p* = .667, η_*p*_^2^ < .01, BF_01_ = 3.22]. The effect of RSI also did not differ by media multitasking group [*F*(1, 68) = 0.91, *p* = .345, η_*p*_^2^ = .01, BF_01_ = 2.78]. In error rate, there were significantly fewer errors after a 100 ms RSI (*M* = 2.80%, *SE* = 0.28%) than a 1,000 ms RSI (*M* = 3.34%, *SE* = 0.30%), *F*(1, 68) = 5.56, *p* = .021, η_*p*_^2^ = .08, BF_01_ = 0.46. There was no effect of media multitasking group, *F*(1, 68) = 0.24, *p* = .626, η_*p*_^2^ < .01, BF_01_ = 3.87, nor did the effect of RSI differ by media multitasking group, *F*(1, 68) = 0.31, *p* = .579, η_*p*_^2^ = .01, BF_01_ = 3.72.

In the odd/even task, reaction time also did not differ by RSI [*F*(1, 68) = 1.06, *p* = .307, η_*p*_^2^ = .02, BF_01_ = 3.26], or media multitasking group[*F*(1, 68) < 0.01, *p* = .948, η_*p*_^2^ < .01, BF_01_ = 3.66], and there was no interaction between RSI and group, *F*(1, 68) = 0.93, *p* = .337, η_*p*_^2^ = .01, BF_01_ = 2.81. Consistent with reaction time, error rate did not significantly differ by RSI [*F*(1, 68) = 2.02, *p* = .160, η_*p*_^2^ = .03, BF_01_ = 1.98] or media multitasking group (*F*(1, 68) = 0.01, *p* = .963, η_*p*_^2^ < .01, BF_01_ = 4.63) nor was there an interaction between RSI and media multitasking group, *F*(1, 68) = 0.09, *p* = .763, η_*p*_^2^ < .01, BF_01_ = 4.11.

#### Voluntary Task Switching

For both reaction time and error rate, we ran separate 2 × 2 × 2 mixed effects ANOVAs with the between-subjects factor of media multitasking group (HMM and LMM) and within-subjects factors of RSI (100 and 1,000 ms) and trial type (repeat and switch).

**Figure 2:**
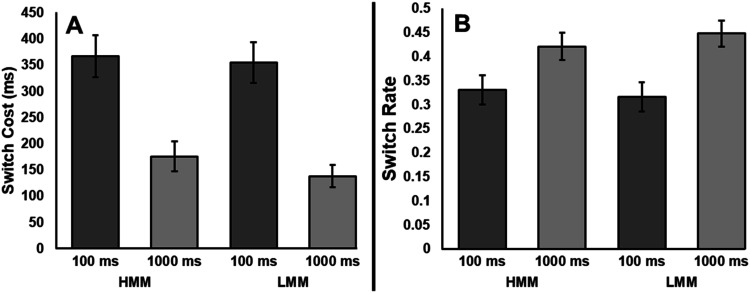
Experiment 2 reaction time switch costs and switch rates after short and long RSIs. Error bars depict standard error. Switch costs were significantly smaller, and switch rates were significantly higher after a long RSI. However, no differences emerged between media multitasking groups.

Participants were significantly faster on task repeats (*M* = 556, *SE* = 18) than task switches (*M* = 815, *SE* = 22), *F*(1, 68) = 173.34, *p* < .001, η_*p*_^2^ = .72, BF_01_ < .001. Participants responded significantly slower after a 100 ms RSI (*M* = 731, *SE* = 20) than a 1,000 ms RSI (*M* = 640, *SE* = 17), *F*(1, 68) = 66.20, *p* < .001, η_*p*_^2^ = .49, BF_01_ < .001. That difference was driven by an interaction between trial type and RSI, such that the switch cost was larger after the 100 ms RSI (*M* = 361, *SE* = 28) than the 1,000 ms RSI (*M* = 158, *SE* = 18), *F*(1, 68) = 60.71, *p* < .001, η_*p*_^2^ = .47, BF_01_ < .001. HMMs and LMMs did not differ from each other significantly in overall reaction time, *F*(1, 68) = 1.45, *p* = .233, η_*p*_^2^ = .02, BF_01_ = 2.24. Moreover, group performance did not significantly differ by RSI (*F*(1, 68) = 0.03, *p* = .866, η_*p*_^2^ < .01, BF_01_ = 5.42) or trial type (*F*(1, 68) = 0.40, *p* = .529, η_*p*_^2^ < .01, BF_01_ = 3.80). Critically, there was also no three-way interaction between RSI, trial type, and media multitasking group, *F*(1, 68) = 0.24, *p* = .627, η_*p*_^2^ < .01, BF_01_ = 3.47 (see Figure 2).

Consistent with reaction time, participants made fewer errors on repeat trials (*M* = 3.04%, *SE* = 0.33%) than switch trials (*M* = 4.41%, *SE* = 0.61%), *F*(1, 68) = 9.73, *p* = .003, η_*p*_^2^ = .13, BF_01_ = 0.07. While there was no effect of RSI, (*F*(1, 68) = 0.41, *p* = .523, η_*p*_^2^ = .01, BF_01_ = 5.78), the switch cost was larger after a 100 ms RSI (*M* = 2.22%, *SE* = 0.68%) than a 1,000 ms RSI (*M* = 0.50%, *SE* = 0.37%), *F*(1, 68) = 7.04, *p* = .010, η_*p*_^2^ = .09, BF_01_ = .13. Paralleling reaction time, error rate did not differ between HMMs and LMMs, *F*(1, 68) = 0.07, *p* = .790, η_*p*_^2^ < .01, BF_01_ = 3.97. Additionally, media multitasking group did not interact with RSI, (*F*(1, 68) = 1.05, *p* = .310, η_*p*_^2^ = .02, BF_01_ = 3.09) or trial type (*F*(1, 68) = 0.25, *p* = .620, η_*p*_^2^ < .01, BF_01_ = 3.84) nor was there an interaction between RSI, trial type, and media multitasking group, *F*(1, 68) = 0.07, *p* = .795, η_*p*_^2^ < .01, BF_01_ = 3.84.

For switch rate, participants switch rate was significantly lower than 50% in both the 100 ms RSI condition (*M* = 31.87%, *SE* = 2.13%; *t*(69) = 8.26, *p* < .001, *d* = .99, BF_01_ < .001) and 1,000 ms RSI condition (*M* = 43.24%, *SE* = 1.99%; *t*(69) = 3.36, *p* = .001, *d* = .40, BF_01_ < .001). To investigate whether media multitasking or RSI affected switch rate, we ran a 2 × 2 mixed effects ANOVA with a between-subjects factor of media multitasking group (HMM and LMM), and a within-subjects factor of RSI (100 and 1,000 ms). Switch rate was significantly lower in the 100 ms condition than the 1,000 ms RSI condition, *F*(1, 68) = 87.99, *p* < .001, η_*p*_^2^ = .56, BF_01_ < .001. However, switch rate did not significantly differ by media multitasking group, *F*(1, 68) = 0.04, *p* = .840, η_*p*_^2^ < .01, BF_01_ = 2.14. While the difference in switch rates between 100 and 1,000 ms RSI was nominally smaller for HMMs (*M* = 9.43%, *SE* = 1.39%) than LMMs (*M* = 13.55%, *SE* = 2.12%), there was not a significant interaction between media multitasking group and RSI, *F*(1, 68) = 2.83, *p* = .097, η_*p*_^2^ = .04, BF_01_ = 1.31 (see Figure 2). Importantly, switch rates did not differ significantly between HMMs and LMMs at 100 ms [*t*(68) = 0.30, *p* = .766, *d* = .07, BF_01_ = 3.91] nor 1,000 ms [*t*(68) = 0.72, *p* = .472, *d* = .17, BF_01_ = 3.25].

### Discussion

While switch rates were higher and switch costs were lower after a long RSI, no difference emerged between HMMs and LMMs for either RSI condition. While this research conceptually replicates previous work that showed that raising the RSI reduces switch costs as well as increasing switch rate (e.g., Arrington, 2008; Arrington & Logan, 2004, 2005; Demanet & Liefooghe, 2014; Liefooghe et al., 2010), we did not see any evidence that VTS is related to media multitasking. Because VTS implicates more internally driven switches, the differences between HMMs and LMMs plausibly relate more strongly to exogenously cued task switching. We discuss this possibility further below.

## General Discussion

In Experiment 1, we found that HMMs switched more effectively than LMMs when cued, but no difference emerged in VTS for switch rate or switch costs in either experiment. Though our RSI manipulation in Experiment 2 increased switch rate and decreased switch costs, the degree to which RSI affected those factors did not differ between HMMs and LMMs. Taken together, our results suggest that media multitasking does relate to the elements of task switching ability captured by CTS, but that relationship does not extend to the task switching behaviors captured by VTS.

A critical question remains: why did a difference between high and low media multitaskers emerge in CTS but not VTS? First, we must understand which elements of task switching are being differently captured by cued and voluntary paradigms. One interpretation of our data is that the internally driven mechanisms in the VTS do not relate to media multitasking in the same way as the more externally driven CTS. However, stimulus-based switching (e.g., Mayr & Bell, 2006) challenges the purely endogenous nature of VTS, and our HMMs and LMMs did not differ in terms of stimulus-based switching (see ESM 1 for a full report). A possibly important distinction to make is between task-irrelevant exogenous cues (e.g., the stimulus switching on the screen) and task-relevant exogenous cues (e.g., the cue in a CTS paradigm). Only the task-relevant exogenous cue provided top-down activation of the task goal and drove differences between HMMs and LMMs, and that element of task switching may be what uniquely relates to media multitasking.

One should also consider the role of preparation of a task set when comparing performance in CTS and VTS paradigms in the current study. Previous work has established that advanced preparation of a task set, rather than the passive decay of the task set on a previous task was sensitive to media multitasking differences (Alzahabi et al., 2017), and VTS typically implicates internal selection and preparation of a task set (Arrington et al., 2014). Because our CTS paradigm made use of a predictable switch pattern (i.e., an AABB pattern), participants plausibly could have internally prepared task sets in CTS as well. If internal preparation of task sets before stimulus (and cue) onset is present in both paradigms, the sole difference between media multitasking groups in the current study may be sensitivity to goal-relevant exogenous cues.

As the impact of stimulus switching on VTS performance implicates environmental influence, we cannot conclude from our results that HMMs and LMMs differed in this respect. In contrast, previous research suggests that HMMs are more susceptible to allowing irrelevant environmental influences into working memory in an AX-CPT task and a filtering task (Ophir et al., 2009). We did not corroborate this effect per se, though it should be noted that the effects reported by Ophir et al. (2009) relate to distractions from an irrelevant stimulus rather than changes in the target stimulus. Second, real world media multitasking is driven by both externally and internally driven switches, but no research has demonstrated whether media multitasking is primarily exogenously or endogenously cued. Without that research, we cannot conclude that differences in media multitasking behavior drove the difference in our results. Relatedly, recent studies have also tried to use more ecologically valid assessments of task switching (Szumowska et al., 2018) and even media multitasking (Lopez & Orr, 2022; Ralph et al., 2014; Seddon et al., 2021). Continuing work in these lines may lead to a better understanding of how media multitasking relates to task switching. For our current study, we can conclude that HMMs switched more effectively than LMMs when switching is based on task-relevant external cues, but not task-irrelevant external cues or internally driven choices.

### Limitations and Future Directions

One may wonder whether the use of the MMES, rather than the MMI, to split high and low media multitasking groups was important in observing our results or weakens the comparison to the existing literature. While this design choice may be a potential limitation, we do not believe it was a pernicious issue for two key reasons. First, our high and low media multitasking groups were also clearly distinct from each other according to the MMI. HMMs had significantly higher MMI scores than LMMs in both experiments, and the effect size was large. Second, the media multitasking and task switching literature is far from uniform in terms of how to create media multitasking groups based on their MMI scores. Studies have split media multitaskers using a criterion of one *SD* higher or lower than the mean (Lui & Wong, 2012; Ophir et al., 2009), using the cutoffs from a previous media multitasking study (Minear et al., 2013), using top and bottom deciles of the collected sample (Baumgartner et al., 2014), or top and bottom quartiles (Alzahabi & Becker, 2013). Our method of classifying HMMs and LMMs is not deviating from a universal standard. That said, future work should consider whether disparate results in media multitasking relate, in some part, to how media multitasking is measured.

An important consideration for this study and the field of media multitasking research more generally is whether the current media multitasking measures (i.e., MMES, MMI) are assessing one behavior (media multitasking) or several different behaviors that are being grouped under the umbrella term of media multitasking. That is, distinct media combinations may represent distinct behaviors as not all types of media multitasking produce the same demand on cognition. More specifically, the same score on either of the media multitasking measures (MMI, our MMES) could be produced despite a person engaging with very different combinations of media. An important step towards capturing media multitasking and its relationship to other cognitive behaviors is to specify the potential subtypes of media multitasking. Certain types of media combinations are more likely than others (Baumgartner & Wiradhany, 2022; Wiradhany & Baumgartner, 2019). Moreover, the frequency of engagement with a particular type of media has been shown to relate to task switching; participants who reported higher action-based video games switched more effectively than participants who did not (Cardoso-Leite et al., 2016). It would be an important theoretical step to go beyond whether task switching relates to media multitasking overall and instead try to understand which elements of media multitasking relate to task switching behavior.

An important design choice that plausibly affected our results (and may help explain variable results across the media multitasking and task switching literature) relates to how we assessed high and low media multitasking groups. As in several previous studies of media multitasking and cognitive ability (e.g., Alzahabi & Becker, 2013; Minear et al., 2013; Ophir et al., 2009) we used an “extreme groups” design to assess differences between HMMs and LMMs. While this approach allows for the assessment of high and low media multitasking behavior while conserving laboratory resources, it may not lead to the most complete understanding of the relationship between media multitasking and task switching behavior. First, the design does not assess the cognitive abilities of people who media multitask an intermediate amount. This group may be valuable to assess as they represent the typical media multitasker. Moreover, we may not accurately capture the true nature of the relationship by ignoring intermediate multitaskers. One study found no relationship between media multitasking and task switching in adolescents when assessed continuously, but that HMMs showed faster task shifting when assessed using extreme groups (Rogobete et al., 2021; cf. Murphy & Shin, 2022, where no relationship was observed between cognitive flexibility and media multitasking when media multitasking was considered continuously). Second, by grouping all HMMs together and all LMMs together, an extreme groups design treats those groups as homogenous rather than containing a range of engagement with media multitasking. Other studies (e.g., Alzahabi et al., 2017; Elbe et al., 2019; Ralph & Smilek, 2017; Schneider & Chun, 2021) have used designs that treat media multitasking as a continuous variable, and future work should consider a continuous approach to better understand the relationship of media multitasking and VTS.

### Conclusion

By using VTS, we assessed elements of task switching that were not captured by previous CTS studies. We first found that HMMs had smaller switch costs than LMMs in CTS, but no difference in switch costs or switch rate emerged in VTS. Moreover, stimulus-based switches did not differentially influence task switching between media multitasking groups. Based on these results, there is evidence that task-relevant exogenously driven switches relate to media multitasking, but endogenously driven and task-irrelevant exogenously driven cues do not relate to media multitasking. We were motivated in part to use VTS to capture the internally driven elements of real-world media multitasking behavior. Although we did not find a relationship between VTS and media multitasking, future research should aim to better assess elements of task switching that relate to real-world media multitasking behavior. Further exploration of these elements will lead to a more complete understanding of the relationship between media multitasking and task switching.

## Electronic Supplementary Materials

The electronic supplementary materials are available with the online version of the article at https://doi.org/10.1027/1618-3169/a000639

**ESM 1.** Task Instructions; Stimulus Repetition Analysis; Media Multitasking Engagement Survey.

## Footnotes

^1^
In the most recent of these reviews, a meta-analysis was performed on both self-report and performance measures of cognitive ability (Parry & le Roux, 2021). In general, the relationship between media multitasking and cognitive performance was small and effect size and the direction of effects was heterogenous in nature. Media multitasking was weakly associated with working memory performance and nonsignificantly associated with performance measures of inhibitory control, sustained attention, interference management, and task management (Parry & le Roux, 2021). Specifically to task switching, these results indicate a potentially heterogenous literature for reasons regarding the measurement of media multitasking and the nature of the task switching paradigms used.

^2^
The MMES was validated in a pilot study and related closely to the MMI. See ESM 1 for the survey, scoring details on the MMES, details on a validation pilot study, and justification for the potential benefits of a new measure of media multitasking. Data for the validation study are available at (https://osf.io/n9b5v/files/). In short, the MMES is appreciably shorter and less cumbersome in terms of number of questions, indexes more recent media types than the MMI, and addresses the absolute time in hours media multitasking rather than a proportion of time.

^3^
Effects were compared across all models. The baseline prior was non-informative and used the following specifications: *r* scale fixed effects = .5, *r* scale random effects = 1, *r* scale covariates = .354. The reported BF_01_ for each main effect was derived from comparing a model with both main effects (i.e., media multitasking group + trial type) to a model without the main effect of interest (e.g., just media multitasking group for the effect of trial type). The reported BF_01_ for the interaction effect was derived from comparing the model with both main effects and the interaction (i.e., media multitasking group + trial type + media multitasking group * trial type) to a model that had just the main effects (i.e., media multitasking group + trial type).

## Notes

**Open Science:** The article includes a DOI generated by a public data repository pointing to the raw data underlying the findings reported in the article.

Open Analytic Code: The analysis script is available online at https://osf.io/n9b5v/ (Colvett, 2025).

Open Data: The data are available online at https://osf.io/n9b5v/ (Colvett, 2025).

Preregistration: The authors have not preregistered this research.
