# Supplementary material for: High and Low Media Multitaskers Differ on Cued But Not Voluntary Task Switching
Source: Exp Psychol. 2025 Apr 23;72(1):1–13. doi: 10.1027/1618-3169/a000639 (PMC12231114; doi:10.1027/1618-3169/a000639)
Supplement: Supplementary file 1 [file zea_72_1_1_esm1.pdf]

https://doi.org/10.1027/1618-3169/a000639

Supplementary Materials

High/ Low Task Instructions:

The goal for this first task is to decide whether a presented number is lower or higher than 3. If the number is lower than 3, press the yellow "Lower" key on the keyboard. If the number is higher than 3, press the green "Higher" key on the keyboard. Press the yellow key to continue.

Once the number appears on the screen, make your decision as quickly as possible. The next number will not appear until you have made a response. Press the green key to continue.

If you are ready to proceed to the practice trials, place your left middle and index fingers on the yellow "Lower" and green "Higher" keys. Press the yellow key to begin the practice trials.

Even/Odd Task Instructions:

You will now be doing a different task. The goal for this task is to decide whether a presented number is even or odd. If the number is even, press the red "Even" key. If the number is odd, press the blue "Odd" key. Press the red key to continue.

Once the number appears on the screen, make your decision as quickly as possible. The next number will not appear until you have made a response. Press the blue key to continue.

If you are ready to proceed to the practice trials, place your right index and middle fingers on the red "Even" key and the blue "Odd" key. Press the red key to begin the practice trials.

https://doi.org/10.1027/1618-3169/a000639

Stimulus Repetition Analysis

Experiment 1

To investigate whether HMMs and LMMs task switching performance was differentially sensitive to task-irrelevant exogenous cues, we assessed the role of stimulus changes in voluntary task switching. For switch cost in both reaction time and error rate, we ran a 2 x 2 x 2 mixed effects ANOVA with a between-subjects factor of media multitasking group (HMM and LMM), and within-subjects factors of trial type (switch and repeat) and stimulus type (change and repetition). Due to concerns with statistical power, we only included participants who had at least 5 observations in each cell of this analysis. Of our original 44 participants, 36 remained (20 HMMs and 16 LMMs).

Participants responded significantly faster when the stimulus repeated (*M* = 615, *SE* = 42) than when the stimulus changed (*M* = 911, *SE* = 36), $F(1, 34) = 252.80$, $p < .001$, $\eta_p^2 = .88$. Stimulus repetition interacted with trial type ($F(1, 34) = 23.65$, $p < .001$, $\eta_p^2 = .41$), such that the switch cost was smaller for stimulus changes (*M* = 191, *SE* = 35) than stimulus repetitions (*M* = 352, *SE* = 38). However, the difference between stimulus repetitions and changes did not differ by media multitasking group, $F(1, 34) = 0.03$, $p = .873$, $\eta_p^2 < .01$, and there was no interaction between media multitasking group, trial type, and stimulus type, $F(1, 34) = 2.19$, $p = .148$, $\eta_p^2 = .06$.

In error rate, participants made significantly fewer errors on stimulus repetitions (*M* = 3.99%, *SE* = 1.02%) than stimulus changes (*M* = 6.39%, *SE* = 1.05%), $F(1, 34) = 6.45$, $p = .016$, $\eta_p^2 = .16$. Paralleling reaction time, there was a significant interaction between trial type and stimulus type, $F(1, 34) = 16.13$, $p < .001$, $\eta_p^2 = .32$, such that the switch cost was smaller when for stimulus changes (*M* = -1.33%, *SE* = 1.08%) than stimulus repetitions (*M* = 5.69%, *SE* =

https://doi.org/10.1027/1618-3169/a000639

1.22%). However, there was no interaction between media multitasking group and stimulus type, $F(1, 34) = 0.33$, $p = .569$, $\eta_p^2 = .01$, nor was there an interaction between media multitasking group, trial type, and stimulus type, $F(1, 34) = 1.15$, $p = .290$, $\eta_p^2 = .03$.

To assess the effect of stimulus switching on switch rate, we ran a 2 x 2 mixed effects ANOVA with a between-subjects factor of media multitasking group (HMM and LMM) and within-subject factor of stimulus type (change and repetition). Stimulus type affected switch rate, as switch rate was significantly higher for stimulus changes (*M* = 40.44%, *SE* = 2.25%) than for stimulus repetitions (*M* = 20.29%, *SE* = 2.92%), $F(1, 42) = 84.98$, $p < .001$, $\eta_p^2 = .67$. However, the difference between stimulus changes and repetitions did not differ between HMMs and LMMs, $F(1, 42) = 0.01$, $p = .910$, $\eta_p^2 < .01$.

Experiment 2

As in Experiment 1, we were interested in whether HMMs and LMMs were differently susceptible to bottom-up influences from the stimulus in VTS. For switch cost, we ran a 2 x 2 x 2 x 2 mixed effects ANOVA with a between-subjects factor of media multitasking group, and within-subjects factors of RSI (100 ms and 1000 ms), trial type (switch and repeat) and stimulus type (change and repetition). Due to concerns with statistical power, we only included participants who had at least 5 observations in each cell of this analysis. Of our original 70 participants, 44 remained (25 HMMs, 19 LMMs). We report here only stimulus type and its interaction with other factors, as the other results are redundant with reports of switch costs.

Participants were significantly faster for stimulus repetitions (*M* = 585, *SE* = 31) than for stimulus changes (*M* = 837, *SE* = 34), $F(1, 42) = 142.35$, $p < .001$, $\eta_p^2 = .77$. Stimulus type interacted with trial type ($F(1, 42) = 13.46$, $p < .001$, $\eta_p^2 = .24$) such that the switch cost was significantly smaller for stimulus changes (*M* = 132, *SE* = 23) than for stimulus repetitions (*M* =

https://doi.org/10.1027/1618-3169/a000639

213, *SE* = 33). Stimulus type did not interact with RSI (*F*(1, 42) = 1.16, *p* = .288, η_p^2 = .03) or media multitasking group (*F*(1, 42) = 0.44, *p* = .510, η_p^2 = .01). There was a significant interaction of stimulus type, trial type, and RSI (*F*(1, 42) = 25.53, *p* < .001, η_p^2 = .24) such that the difference between switch costs for stimulus repetitions and changes was larger at 100 RSI (*M* = 170, *SE* = 29) than at 1000 RSI (M= -8, SE = 26). However, there was no interaction of stimulus type, trial type, and media multitasking group, *F*(1, 42) = 2.26, *p* = .141, η_p^2 = .05, nor was there an interaction of stimulus type, RSI, and media multitasking group, *F*(1, 42) = 0.01, *p* = .932, η_p^2 < .01. Finally, there was no interaction between trial type, RSI, stimulus type, and media multitasking group *F*(1, 42) = 1.35, *p* = .252, η_p^2 = .03.

Consistent with reaction time, participants were significantly more accurate for stimulus repetitions (*M* = 2.56%, *SE* = 0.67%) than for stimulus changes (*M* = 4.72%, *SE* = 0.80%), *F*(1, 42) = 14.84, *p* < .001, η_p^2 = .26. Additionally, switch cost was smaller for stimulus changes (*M* = -0.17%, *SE* = 0.95%) than for stimulus repetitions (*M* = 2.85%, *SE* = 0.89%), *F*(1, 42) = 10.96, *p* = .002, η_p^2 = .21. However, stimulus type did not interact with RSI (*F*(1, 42) = 1.80, *p* = .187, η_p^2 = .04), or with media multitasking group (*F*(1, 42) = 0.22, *p* = .644, η_p^2 = .01). There was a not an interaction of stimulus type, trial type, and RSI, *F*(1, 42) = 0.16, *p* = .688, η_p^2 < .01, nor was there an interaction of stimulus type, trial type, and media multitasking group, *F*(1, 42) = 0.07, *p* = .800, η_p^2 < .01. There was a non-significant interaction of stimulus type, RSI, and media multitasking group, *F*(1, 42) = 3.95, *p* = .053, η_p^2 = .09. Finally, there was a non-significant interaction between trial type, RSI, stimulus type, and media multitasking group *F*(1, 42) = 2.94, *p* = .094, η_p^2 = .07.

For switch rate, we ran a 2 x 2 x 2 mixed effect ANOVA with a between-subjects factor of media multitasking group (HMM and LMM), and within-subjects factors of RSI (100 ms and

https://doi.org/10.1027/1618-3169/a000639

1000 ms) and stimulus type (change and repetition). Switch rates were significantly higher in the 1000 ms RSI condition (*M* = 42.24%, *SE* = 1.99%) than the 100 ms RSI condition (*M* = 31.87%, *SE* = 2.13%), $F(1, 68) = 88.58$, $p < .001$, $\eta_p^2 = .57$. Switch rate was significantly lower on stimulus repetitions (*M* = 30.58%, *SE* = 2.72%) than on stimulus changes (*M* = 44.86%, *SE* = 2.02%), $F(1, 68) = 62.69$, $p < .001$, $\eta_p^2 = .48$. RSI and stimulus type interacted ($F(1, 68) = 12.01$, $p < .001$, $\eta_p^2 = .15$), such that the difference between repeat and stimulus types was larger in the 100 ms condition (*M* = 17.45%, *SE* = 2.30%) than in the 1000 ms condition (*M* = 11.11%, *SE* = 1.77%). There was no effect of media multitasking group, $F(1, 68) = 0.05$, $p = .821$, $\eta_p^2 < .01$. Finally, media multitasking group did not interact with stimulus type ($F(1, 68) = 0.48$, $p = .491$, $\eta_p^2 = .01$), RSI ($F(1, 68) = 2.77$, $p = .100$, $\eta_p^2 = .04$) or RSI and stimulus type, ($F(1, 68) = 0.14$, $p = .713$, $\eta_p^2 < .01$).

https://doi.org/10.1027/1618-3169/a000639

Media Multitasking Engagement Survey

The MMES was created to assess media multitasking in a format that was easier and more efficient for participants to fill out and up to date in terms of what forms of media were surveyed Critically, the MMES indexed media multitasking as the *absolute time in hours* spent daily multitasking with media. The survey was adapted in part from the media multitasking measure used by Xu, Wang, and David (2016). The survey was designed to determine the approximate amount of time spent with 15 types of media on a typical day and estimates the amount of time spent media multitasking in hours with each type of media. The survey consists of only 30 questions, appreciably shorter than the MMI. Additionally, the MMES assesses relatively more recent types of media that the MMI does not, such as social media and video chatting.

The MMES was validated in a pilot study and related closely to the MMI. The validation study asked a separate sample of 213 Wake Forest University undergraduates (120 Males, 92 Females; Age M = 18.82 years, SD = .89) to fill out a series of questionnaires in groups of 10-30 individuals in a classroom setting. MMES scores correlated positively with MMI scores, $r(153) = .50$, $p < .001$ (See Supplemental Table 1). Note that we did not include data from participants who did not answer all questions of the survey. While 203 participants had complete, usable data for the MMES, we found only 165 participants had usable data from the MMI. This difference lends credence to the idea that the MMI is a cumbersome measure that is hard for participants to use. This difference is possibly pernicious, as the researcher may unintentionally exclude the type of participant who would not complete the survey. Data for this validation study is available at (https://osf.io/n9b5v/files/).

https://doi.org/10.1027/1618-3169/a000639

Supplemental Table 1

Correlations between Media Multitasking Measures and Important Behavioral Variables

	MMI	*MMES*	*BIS*	*NFC*	*BPS*	*Distraction*	*Attention Shift*	*ARCES*
MMI	*r* = 1<br>*n* = 165	*r* = .50**<br>*n* = 155	*r* = .22**<br>*n* = 153	*r* = .08<br>*n* = 159	*r* = .17*<br>*n* = 157	*r* = .02<br>*n* = 163	*r* = .07<br>*n* = 164	*r* = .41**<br>*n* = 163
MMES		*r* = 1<br>*n* = 203	*r* = .15*<br>*n* = 192	*r* = -.02<br>*n* =196	*r* = .19**<br>*n* = 195	*r* = -.02<br>*n* = 200	*r* = .06<br>*n* = 201	*r* = .23**<br>*n* = 201
BIS			*r* = 1<br>*n* = 200	*r* = .38**<br>*n* = 194	*r* = .40**<br>*n* = 194	*r* = .28**<br>*n* = 197	*r* = .43**<br>*n* = 199	*r* = .47**<br>*n* = 199
NFC				*r* = 1<br>*n* = 206	*r* = .36**<br>*n* = 198	*r* = .26**<br>*n* = 203	*r* = .30**<br>*n* = 204	*r* = .12<br>*n* = 205
BPS					*r* = 1<br>*n* = 203	*r* = .29**<br>*n* = 200	*r* = .51**<br>*n* = 201	*r* = .26**<br>*n* = 202
Distraction						*r* = 1<br>*n* = 210	*r* = .45**<br>*n* =209	*r* = .32**<br>*n* = 208
Attention Shift							*r* = 1<br>*n* =211	*r* = .30**<br>*n* = 209
ARCES								*r* = 1<br>*n* = 212

Note: All reported correlations are two-tailed. One asterisk symbolizes that the p-value for the correlation is less than .05. Two asterisks symbolizes that the p-value for the correlation is less than .01. One correlation of note is that the MMES correlates strongly and positively with the MMI. Moreover, the MMES and the MMI show the same pattern of correlations with the other

https://doi.org/10.1027/1618-3169/a000639

behavioral variables. The MMES demonstrates strong convergent validity and discriminant validity with the MMI.

These other measures used were as follows:

The Attention Related Cognitive Errors Scale (ARCES; Carriere, Cheyne, & Smilek, 2008) is a 12-item measure of everyday mistakes that people make as a result of not paying sufficient attention to the task at hand. Participants are asked how often they do a particular action, and responses are given on a scale of 1 (never) to 5 (very often). An example question is: “I have lost track of a conversation because I zoned out when someone else was talking.” Participants’ scores are the sums of their responses across all the questions.

Barrett’s Impulsivity Scale (BIS-11; Patton & Stanford, 1995) is a 30-item measure designed to assess the behavioral construct of impulsiveness. Participants are asked how often they do a particular action, and responses are given on a scale of 1 (Rarely/Never) to 4 (Almost Always/Always). Example questions include: “I plan trips well ahead of time” and “I am a steady thinker.” Participants’ scores are the sums of their responses across all the questions.

The Need for Cognition Scale (NFC; Cacioppo & Petty, 1982) is an 18-item measure that assesses how much enjoyment an individual gets from engaging in deep thought. Participants are asked to read the description of a behavior and rate how characteristic the behavior is of them on a scale of 1 (extremely characteristic) to 5 (extremely uncharacteristic). An example question is: “I only think as hard as I have to.” Participants’ scores are the sum of their responses across all the questions.

The Boredom Proneness Scale (BPS; Farmer & Sundberg, 1982) is a 28-item measure designed to assess how easily an individual gets bored. Participants are asked to read the description of a behavior and rate the degree to which they agree that the description describes

https://doi.org/10.1027/1618-3169/a000639

them on a scale of 1 (highly disagree) to 7 (highly agree). An example question is: "I find it easy to entertain myself." Participants' scores are the sum of their responses across all the questions.

The Distractibility and Attention Shifting Scale (Carreire et al., 2013) is an 8-item measure designed to assess the attentional control of a participant as it relates to being distracted and shifting attention between tasks. Participants are asked to read the description of a behavior and rate how often they do the behavior on a scale of 1 (Almost Never) to 5 (Always). Example questions include: "It is difficult for me to alternate between two different tasks" and "It's very hard for me to concentrate on a difficult task when there are noises around." Participants' scores are the sums of their responses across all the questions.

The Demographics questionnaire is a questionnaire designed for this study. This measure asks participants to indicate their gender, age, year in school, whether they have an ADD/ADHD diagnosis, and if they are currently taking medication for ADD/ADHD.

There are two key distinctions between the MMES and MMI that allows the MMES to assess media multitasking behavior more easily and potentially capture distinct elements of media multitasking behavior. First, the MMES consists of only 30 questions, appreciably shorter than the MMI. The longer length of the MMI (144 questions, all of which must be filled out to obtain a score) may be difficult for participants, leading to possible issues with fatigue, confusion, or missed responses. Moreover, it is possible that the cognitive and media multitasking profile of participants who do not complete the survey may differ from those who do. Second, it has been noted that the MMI assesses the *proportion* of media time that is spent in media multitasking rather than *total hours* spent media multitasking (see Alzahabi & Becker, 2013). Someone who spends two hours with media and one hour of that time media multitasking would have a similar score to someone who spends 40 hours with media and media multitasks

https://doi.org/10.1027/1618-3169/a000639

for 20 of those hours. Despite similar MMI scores, those participants plausibly have qualitatively different media multitasking behaviors. The MMES instead indexes media multitasking as the *absolute time in hours* spent daily multitasking with media.

https://doi.org/10.1027/1618-3169/a000639

Media Multitasking Engagement Survey

SCORING INSTRUCTIONS NOT GIVEN TO PARTICIPANTS: There are 15 types of media being inventoried. For each media, participants are asked how many hours they spend with the media ("Media Hours"), and the percentage of time they are using that media as well as some other form of media ("Percent Multitasking").

For hours, participants answered a number 1-7. 1 should be recoded into 0, 2 should be recoded into .5, 3 should be recoded into 1.5, 4 should be recoded into 2.5, 5 should be recoded into 3.5, 6 should be recoded into 4.5, and 7 should be recoded to 5.5. In the multitasking section, negatives and blanks should be recoded to 0.

For each media, a variable called "Multitasking Hours" should be created by multiplying Media Hours for a particular media by Percent Multitasking with that media. For example, if a participant spent 2 hours a day reading, and 25% of that time multitasking, their Multitasking Hours are .5 for Reading.

"Total Media Hours" should be calculated by adding together the Media Hours for each of the 15 media types. "Media Multitasking Engagement Score" should be calculated by adding together the Multitasking Hours for each of the 15 subtypes.

A variable called "Multitasking Quotient" can also calculated by dividing Multitasking Hours by Total Media Hours. This gives us a number between 0 and 1 that represents amount of media time spent multitasking.

INSTRUCTIONS FOR PARTICIPANTS: The next questionnaire assesses how an individual engages with media. You will be asked to estimate how many hours you spend using a particular type of media on a typical day. A typical day refers to a normal school or work day. This is an estimate of total hours throughout the day, not consecutive hours with one kind of media.

After that, you will be asked how often you use other kinds of media concurrently with that type of media. An example of using media concurrently would be listening to music while reading a book or watching television while writing an email. **If you do not use any kind of media concurrently with the primary media, please move the slider to 0.**

These are the types of media multitasking inventoried:

> watching video content online, television or movies, playing video or computer games, reading (not for work or school), studying or doing homework, email, texting or instant messaging, posting on or using social media, talking on the phone, video chatting, having face-to-face conversations, driving, web-surfing, using a computer used for offline purposes, listening to music, listening to non-music audio

https://doi.org/10.1027/1618-3169/a000639

1. How many hours on a typical day do you spend watching television, movies, or online video content?

Zero
Less than 1 hour
About 1 to 2 hours
About 2 to 3 hours
About 3 to 4 hours
About 4 to 5 hours
More than 5 hours

2. Of the time spent **watching online video content, television or movies**, what percentage of that time is spent using any other kind of media concurrently?

Remember, these are the types of media being surveyed: playing video or computer games; reading; studying or doing homework; email; texting or instant messaging; using social media; talking on the phone or video chatting; having face-to-face conversations; driving; web-surfing or using a computer; listening to music or listening to non-music audio.

[Slider]

3. How many hours on a typical day do you spend playing video or computer games?

Zero
Less than 1 hour
About 1 to 2 hours
About 2 to 3 hours
About 3 to 4 hours
About 4 to 5 hours
More than 5 hours

4. Of the time spent **playing video or computer games**, what percentage of that time is spent using any other kind of media concurrently?

Remember, these are the types of media being surveyed: watching video content online, television, or movies; reading; studying or doing homework; email, texting or instant messaging; using social media; talking on the phone or video chatting; having face-to-face conversations; driving; web-surfing or using a computer; listening to music or listening to non-music audio.

[Slider]

5. How many hours on a typical day do you spend reading (not for work or school)?

Zero
Less than 1 hour
About 1 to 2 hours

https://doi.org/10.1027/1618-3169/a000639

About 2 to 3 hours
About 3 to 4 hours
About 4 to 5 hours
More than 5 hours

6. Of the time spent **reading (not for work or school)**, what percentage of that time is spent using any other kind of media concurrently?

Remember, these are the types of media being surveyed: watching video content online, television, or movies; playing video or computer games; studying or doing homework; email; texting or instant messaging; using social media; talking on the phone or video chatting; having face-to-face conversations; driving; web-surfing or using a computer; listening to music or listening to non-music audio.

[Slider]

7. How many hours on a typical day do you spend studying or doing homework?

Zero
Less than 1 hour
About 1 to 2 hours
About 2 to 3 hours
About 3 to 4 hours
About 4 to 5 hours
More than 5 hours

8. Of the time spent **studying or doing homework**, what percentage of that time is spent using any other kind of media concurrently?

Remember, these are the types of media being surveyed: watching video content online, television, or movies; playing video or computer games; reading; email; texting or instant messaging; using social media; talking on the phone or video chatting; having face-to-face conversations; driving; web-surfing or using a computer; listening to music or listening to non-music audio.

[Slider]

9. How many hours on a typical day do you spend using email?

Zero
Less than 1 hour
About 1 to 2 hours
About 2 to 3 hours
About 3 to 4 hours
About 4 to 5 hours
More than 5 hours

https://doi.org/10.1027/1618-3169/a000639

10. Of the time spent **using email**, what percentage of that time is spent using any other kind of media concurrently?

Remember, these are the types of media being surveyed: watching video content online, television, or movies; playing video or computer games; reading, studying or doing homework; texting or instant messaging; using social media; talking on the phone or video chatting; having face-to-face conversations; driving; web-surfing or using a computer; listening to music or listening to non-music audio.

[Slider]

11. How many hours on a typical day do you spend texting, instant messaging, or messaging on social network?

Zero
Less than 1 hour
About 1 to 2 hours
About 2 to 3 hours
About 3 to 4 hours
About 4 to 5 hours
More than 5 hours

12. Of the time spent **texting, instant messaging, or messaging on social network**, what percentage of that time is spent using any other kind of media concurrently?

Remember, these are the types of media being surveyed: watching video content online, television, or movies, playing video or computer games, reading, studying or doing homework; email; using social media; talking on the phone or video chatting; having face-to-face conversations; driving; web-surfing or using a computer; listening to music or listening to non-music audio.

[Slider]

13. How many hours on a typical day do you spend posting on or using social media?

Zero
Less than 1 hour
About 1 to 2 hours
About 2 to 3 hours
About 3 to 4 hours
About 4 to 5 hours
More than 5 hours

14. Of the time spent **posting on or using social media**, what percentage of that time is spent using any other kind of media concurrently?

https://doi.org/10.1027/1618-3169/a000639

Remember, these are the types of media being surveyed: watching video content online, television, or movies, playing video or computer games, reading, studying or doing homework; email; texting or instant messaging; talking on the phone or video chatting; having face-to-face conversations; driving; web-surfing or using a computer; listening to music or listening to non-music audio.

[Slider]

15. How many hours on a typical day do you spend talking on the phone?

Zero
Less than 1 hour
About 1 to 2 hours
About 2 to 3 hours
About 3 to 4 hours
About 4 to 5 hours
More than 5 hours

16. Of the time spent **talking on the phone**, what percentage of that time is spent using any other kind of media concurrently?

Remember, these are the types of media being surveyed: watching video content online, television, or movies, playing video or computer games, reading, studying or doing homework; email; texting or instant messaging; using social media; video chatting; having face-to-face conversations; driving; web-surfing or using a computer; listening to music or listening to non-music audio.

[Slider]

17. How many hours on a typical day do you spend video chatting, such as Skype or Facetime?

Zero
Less than 1 hour
About 1 to 2 hours
About 2 to 3 hours
About 3 to 4 hours
About 4 to 5 hours
More than 5 hours

18. Of the time spent **video chatting**, what percentage of that time is spent using any other kind of media concurrently?

Remember, these are the types of media being surveyed: watching video content online, television, or movies; playing video or computer games; reading; studying or doing homework; email, texting or instant messaging; using social media; talking on the phone; having face-to-face

https://doi.org/10.1027/1618-3169/a000639

conversations; driving; web-surfing or using a computer; listening to music or listening to non-music audio.

[Slider]

19. How many hours on a typical day do you spend having face-to-face conversations?

Zero
Less than 1 hour
About 1 to 2 hours
About 2 to 3 hours
About 3 to 4 hours
About 4 to 5 hours
More than 5 hours

20. Of the time spent **having face-to-face conversations**, what percentage of that time is spent using any other kind of media concurrently?

Remember, these are the types of media being surveyed: watching video content online, television, or movies; playing video or computer games; reading; studying or doing homework; email; texting or instant messaging; using social media; talking on the phone or video chatting; driving; web-surfing or using a computer; listening to music or listening to non-music audio.

[Slider]

21. How many hours on a typical day do you spend driving?

Zero
Less than 1 hour
About 1 to 2 hours
About 2 to 3 hours
About 3 to 4 hours
About 4 to 5 hours
More than 5 hours

22. Of the time spent **driving**, what percentage of that time is spent using any other kind of media concurrently?

Remember, these are the types of media being surveyed: watching video content online, television, or movies; playing video or computer games; reading; studying or doing homework; email; texting or instant messaging; using social media; talking on the phone or video chatting; having face-to-face conversations; web-surfing or using a computer; listening to music or listening to non-music audio.

[Slider]

https://doi.org/10.1027/1618-3169/a000639

23. How many hours on a typical day do you spend web surfing or with online content?

Zero
Less than 1 hour
About 1 to 2 hours
About 2 to 3 hours
About 3 to 4 hours
About 4 to 5 hours
More than 5 hours

24. Of the time spent **web surfing or with online content**, what percentage of that time is spent using any other kind of media concurrently?

Remember, these are the types of media being surveyed: watching video content online, television, or movies; playing video or computer games; reading; studying or doing homework; email; texting or instant messaging; using social media; talking on the phone or video chatting; having face-to-face conversations; driving; using a computer; listening to music or listening to non-music audio.

[Slider]

25. How many hours on a typical day do you spend using your computer or laptop for non-online purposes, such as word processing?

Zero
Less than 1 hour
About 1 to 2 hours
About 2 to 3 hours
About 3 to 4 hours
About 4 to 5 hours
More than 5 hours

26. Of the time spent **using your computer or laptop for non-online purposes, such as word processing,** what percentage of that time is spent using any other kind of media concurrently?

Remember, these are the types of media being surveyed: watching video content online, television, or movies; playing video or computer games; reading; studying or doing homework; email; texting or instant messaging; using social media; talking on the phone or video chatting; having face-to-face conversations; driving; web-surfing; listening to music or listening to non-music audio.

[Slider]

27. How many hours on a typical day do you spend listening to music?

Zero

https://doi.org/10.1027/1618-3169/a000639

Less than 1 hour
About 1 to 2 hours
About 2 to 3 hours
About 3 to 4 hours
About 4 to 5 hours
More than 5 hours

28. Of the time spent **listening to music**, what percentage of that time is spent using any other kind of media concurrently?

Remember, these are the types of media being surveyed: watching video content online, television, or movies; playing video or computer games; reading; studying or doing homework; email; texting or instant messaging; using social media; talking on the phone or video chatting; having face-to-face conversations; driving; web-surfing or using a computer or listening to non-music audio.

[Slider]

29. How many hours on a typical day do you spend listening to non-music audio, such as podcasts, news, or talk radio?

Zero
Less than 1 hour
About 1 to 2 hours
About 2 to 3 hours
About 3 to 4 hours
About 4 to 5 hours
More than 5 hours

30. Of the time spent **listening to non-music audio** what percentage of that time is spent using any other kind of media concurrently?

Remember, these are the types of media being surveyed: watching video content online, television, or movies; playing video or computer games; reading; studying or doing homework; email; texting or instant messaging; using social media; talking on the phone or video chatting; having face-to-face conversations; driving; web-surfing or using a computer; listening to music.

[Slider]
